# Utilization of Provider–Patient Communication Tools and Approaches to Facilitate Vaccine Confidence: A Systematic Review

**DOI:** 10.3390/vaccines13111121

**Published:** 2025-10-31

**Authors:** Aleda M. H. Chen, Juanita A. Draime, Mia Engert, Aaron Pachucki, Thurein Zan, Eliya Craig, Nathan Gibson, Chukwunonso Chukwuemeka, Stephanie M. Tubb, Justin W. Cole

**Affiliations:** 1 School of Pharmacy, Cedarville University, Cedarville, OH 45314, USA; juanitaadraime@cedarville.edu (J.A.D.); tzan@cedarville.edu (T.Z.); ecraig@cedarville.edu (E.C.); ngibson@cedarville.edu (N.G.); chukwuemekac@cedarville.edu (C.C.); smtubb@cedarville.edu (S.M.T.); jwcole@cedarville.edu (J.W.C.); 2 Department of Psychology, Cedarville University, Cedarville, OH 45314, USA; engertm@cedarville.edu (M.E.); apachucki@cedarville.edu (A.P.)

**Keywords:** vaccines, vaccine acceptance, vaccine hesitancy, vaccine confidence, communication

## Abstract

**Background/Objectives**: With declining vaccine coverage and rising concerns regarding vaccines, vaccine-preventable diseases are rising. Many research studies have examined approaches to enhance the acceptance of vaccines, but the integration of these approaches into practice has been limited. Thus, the objective was to identify recent evidence surrounding provider–patient communication tools and approaches related to vaccination confidence. **Methods**: A systematic review following PRISMA methodology was conducted. Using a pre-specified search strategy aligned with the research objective and performed in PubMed, CINAHL, and Web of Science, articles were evaluated by two researchers independently, with a third resolving discrepancies, at the title and abstract screening, full-text review, quality assessment, and data extraction phases. Extraction data were descriptively analyzed, including the impact on vaccine acceptance, and synthesized thematically. **Results**: From the 2291 studies which underwent screening, a total of, 143 articles were included. Most studies were conducted in the United States, in the outpatient setting, and utilized physicians and nurses to deliver the intervention. Many vaccines were covered, with the greatest number of studies focusing on influenza, HPV, and pneumococcal vaccines. The three predominant communication approaches and tools utilized were provider-focused training, direct patient education/materials, and provider–patient communication strategies. These strategies often focused on addressing knowledge gaps, health beliefs, and common concerns. Over two-thirds of studies increased vaccine acceptance following communication interventions. **Conclusions**: To address vaccine concerns, it is important to ensure providers have the education and training necessary as well as tools to address underlying causes of concerns. When equipped, providers are able to improve vaccine acceptance.

## 1. Introduction

Coverage of vaccines has faced challenges in recent years. Vaccine coverage, already below target levels, has continued to decline, especially among pediatric populations [1,2,3]. Many vaccinations have reduced infectious disease-related morbidity and mortality [4]; this requires substantial coverage of the population to prevent the spread of disease and protect those unable to be vaccinated [5]. Despite is, public confidence in vaccines has waned due to varying factors, including the recent COVID-19 pandemic and beliefs related to COVID vaccines [6,7,8,9]. Thus, many patients and caregivers of children may forgo or delay the receipt of a vaccine(s). Concerns about vaccines often stem from perceived safety and efficacy issues, weak social support, diminished perceptions of benefit, and misinformation [6,10,11,12,13,14,15,16]. Although vaccine confidence has declined, healthcare providers continue to increase vaccine acceptance [17] and are among the most trusted sources of vaccine information [18]. Leveraging this trust through effective communication is critical to addressing public confidence in vaccines.

Because declining vaccine confidence can contribute to increased global burden of disease, healthcare professionals must facilitate open dialogue to elicit underlying health beliefs, address misinformation, and provide evidence-based information regarding vaccine safety and efficacy. Prior work has demonstrated the effectiveness of interventions to enhancing provider–patient communication during vaccine confidence conversations, resulting in increased vaccine uptake [19,20,21,22,23]. For example, pediatric providers who utilized motivational interviewing, a patient-centered, collaborative approach to exploring perspectives and identifying reasons and desire for change, as well as communication tool were able to see a decline in documented vaccine refusals and an increase in seasonal influenza vaccine rates [19]. Expanding this approach into community pharmacies with patients who expressed initial hesitancy resulted in 35.4% of these patients receiving a vaccine [24]. Providers also have seen the benefits of these approaches in maternity patients [23,25].

Improving the implementation of evidence-based practices, such as vaccination confidence provider–patient communication tools and approaches, must include behavioral change by providers. Rather than being directive about the need to vaccinate and accepting the patient’s initial response to vaccinations, providers should facilitate open conversations, elicit patient perspectives, and collaborate with the patient to achieve health goals. However, despite growing evidence of effective communication-based interventions [26,27,28], there is limited integration into everyday practice. Providers often lack structured tools, adequate training, or organizational support to implement these approaches consistently. Thus, the objective of this systematic review was to identify recent evidence surrounding provider–patient communication tools and approaches related to vaccination confidence in order to provide the first steps for translating these findings into practice.

## 2. Materials and Methods

The Preferred Reporting Items for Systematic reviews and Meta-Analyses (PRISMA) guideline was followed for this systematic review [29]. Prior to beginning the systematic review, the study protocol was registered in PROSPERO (CRD42024546568). Due to the heterogeneity of the study designs and outcomes that would be included, the planned analytical approach from the beginning was a qualitative synthesis rather than a meta-analysis.

Inclusion and exclusion criteria were identified. To be included, articles must be peer-reviewed, be primary literature research articles, be available in full-text, written in English, and include provider–patient communication tools/approaches related to vaccination confidence (description of the use of communication tools by a healthcare professional to talk about vaccines with patients or description of the use of communication approaches by a healthcare professional to talk about vaccines with patients). Articles that did not meet those criteria were excluded. Any review articles were excluded, and systematic reviews/meta-analyses were reviewed for any articles missed in the search and then excluded.

A comprehensive search strategy was created in collaboration with a research librarian. The core search strategy included the following terms, which were iterated appropriately depending on the database searched: (healthcare provider OR health personnel OR healthcare workers OR healthcare professional) AND (patient) AND (vaccine) AND (communication OR health communication OR patient education OR health education). PubMed, CINAHL, and Web of Science were utilized to perform the search from 1 January 2000, to date of search (14 May 2024). The search was piloted and refined until tests of the results indicated a comprehensive search. Results from the search were downloaded into Zotero (version 7), a reference management system, for cleaning before uploading into Covidence, a systematic review management system.

Prior to beginning the review process, the research team was trained on the protocol and the Covidence system. At each phase, two researchers performed the step independently, with a third researcher resolving any conflicts. In the first phase, titles and abstracts were reviewed for inclusion, and articles that received a “yes” or “maybe” moved on to full-text review. In the second phase, full-text copies of the articles were obtained and uploaded to Covidence. Full-text articles were reviewed against the inclusion and exclusion criteria. Articles that met these criteria advanced to data extraction. A pre-built template was created in Covidence to pull key article information. The following study characteristics were extracted: article characteristics (setting, study design, study aim, study length, type of provider(s), patient characteristics (pediatric, adolescent, adult, older adult), vaccine(s), type of intervention (communication approach, tool), description of intervention, and key findings. Then, the quality of the study was assessed using the Mixed-Methods Appraisal Tool (MMAT) [30], which allowed for the flexibility of quality assessment given the variety of study designs. Scores could range from 0 to 7, with higher scores indicating greater quality.

Findings from the data extraction were downloaded from Covidence and synthesized into tables. Key findings were thematically drawn from the data extraction tables. Where possible, the findings were categorized and summarized. Outcomes of the communication approach and tool interventions were categorized as: positive (improvement in vaccine acceptance), negative (decline in vaccine acceptance), neutral (limited or no change in vaccine acceptance), or mixed findings (could include positive, negative, and/or neutral in combination).

## 3. Results

**Figure 1:**
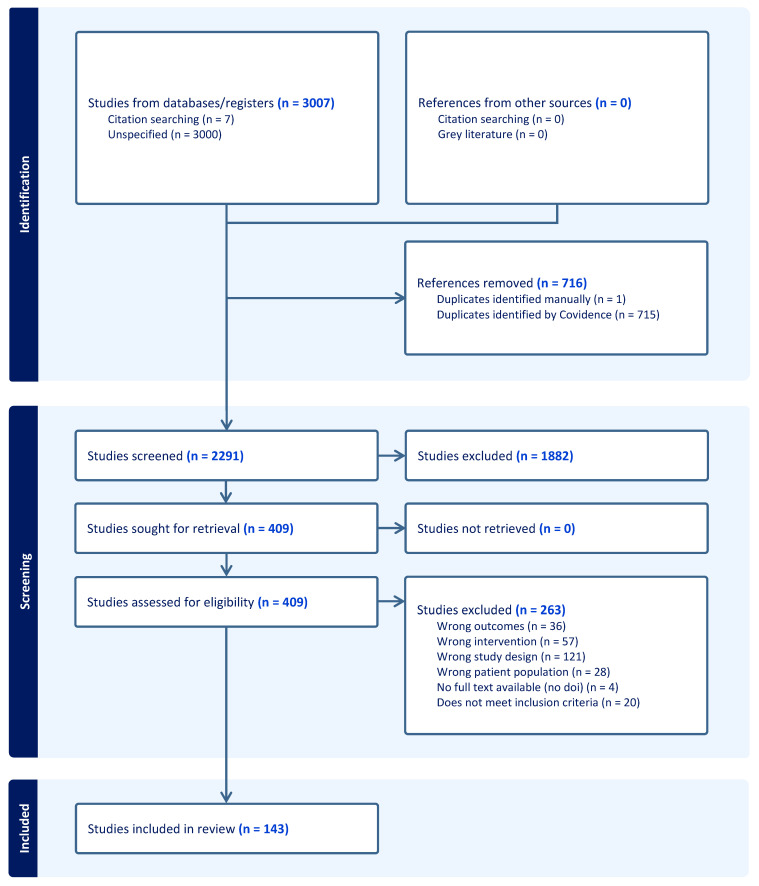
PRISMA Diagram of Article Identification, Screening, and Inclusion.

In Figure 1, the PRISMA diagram shows the article identification process, screening, and inclusion.

From the 2291 studies which underwent screening, a total of 143 articles met inclusion criteria and underwent data extraction [19,22,31,32,33,34,35,36,37,38,39,40,41,42,43,44,45,46,47,48,49,50,51,52,53,54,55,56,57,58,59,60,61,62,63,64,65,66,67,68,69,70,71,72,73,74,75,76,77,78,79,80,81,82,83,84,85,86,87,88,89,90,91,92,93,94,95,96,97,98,99,100,101,102,103,104,105,106,107,108,109,110,111,112,113,114,115,116,117,118,119,120,121,122,123,124,125,126,127,128,129,130,131,132,133,134,135,136,137,138,139,140,141,142,143,144,145,146,147,148,149,150,151,152,153,154,155,156,157,158,159,160,161,162,163,164,165,166,167,168,169,170,171]. Table 1 provides an overview of the studies included, overall and by age group. Most studies were conducted in the United States, in the outpatient setting, and were non-randomized controlled trial (RCT) designs or RCTs. Physicians and nurses most frequently delivered the interventions, which typically included both communication tools and approaches. Nine studies utilized community pharmacies and pharmacists for the intervention. Many vaccines were covered, with the greatest number of studies focusing on influenza, HPV, and pneumococcal vaccines.

Communication approaches and tools utilized are summarized in Table 2. The three predominant approaches were provider-focused training and tools, patient education materials and communication, and provider–patient communication strategies. Many interventions trained providers in a variety of formats to provide information on vaccines as well as best practices in communicating and sharing decision-making about vaccines. These strategies often focused on addressing knowledge gaps, health beliefs, and common concerns. A small portion of the studies trained providers in behavior-related strategies, such as motivational interviewing (MI). Patient-focused education strategies were focused on sharing information with patients about vaccines to dispel myths, reframe perceptions, and summarize key information. Many of the materials were delivered in a written format, but some studies did include media-based interventions and information-based provider interventions. Provider–patient communication strategies included approaches to conducting vaccine conversations, such as “Ask, Acknowledge, Advise” or the presumptive versus participatory approaches of introducing vaccines. Some other strategies focused on behavioral approaches, such as MI or the 4C model of vaccine hesitancy. A few studies also included communication tools to help the providers in utilizing these approaches. System-level and structural supports commonly focused on integrating standing orders for vaccines and prompts in the electronic health record to ask about vaccines. Reminder and recall interventions focused on the use of technology or trained provider extenders (e.g., community health workers, patient navigators) to schedule and remind patients regarding vaccine appointments.

The impact of the tool or approach on vaccines, namely vaccine acceptance, is noted in Table 3.

Two-thirds of studies reported enhanced vaccine acceptance, with more benefits seen in adult studies. Pediatric studies had higher rates of mixed findings with positive and neutral results versus adult studies.

In the most common vaccines studied (HPV, influenza, and pneumococcal), intervention outcomes were overall positive. In HPV vaccination interventions, outcomes were predominantly positive or neutral. Reminder and recall interventions, patient education strategies, and provider–patient communication strategies were consistently used in the HPV interventions, particularly among adolescent patients and their parents. For example, clinics receiving announcement training had a 5.4% increase in HPV vaccine acceptance among 11- and 12-year-olds versus controls [38], while another text message reminder-based intervention improved HPV series completion from 30% to 49% and reduced time to completion by an average of 71 days [127]. A communication-based intervention saw an 17% increase in vaccine initiation and an 18% increase in vaccine completion, with an increase to 31% post-intervention [41].

Studies examining influenza vaccination interventions also revealed predominantly positive effects, with increased vaccine acceptance. Interventions focused on provider–patient communication and reminder/recall. For example, one study documented an increase in immunization rates from 8% to 39% following a free clinic-wide education and outreach initiative [61], while a transplant pharmacist-driven education and intervention initiative reported an increase from 36% to 74% after implementation [68]. Studies assessing interventions to improve pneumococcal vaccination rates consistently demonstrated positive effects and utilized provider–patient communication, system-level supports, and patient education. For example, a patient education initiative in primary care improved vaccine acceptance from 24% pre-intervention to 44% post-intervention [46]. Among liver-transplant recipients, annual pneumococcal vaccine acceptance rates increased from 46% to 58% during the intervention period (reminder/recall intervention and a provider communication tool) [54]. Similarly, 62% of intervention-group participants with inflammatory bowel disease accepted the pneumococcal vaccine compared with 23% in the control group. The intervention consisted of nurse-led communication (patient-provider communication) and patient educational materials [47].

The average quality assessment of the included studies was 6.03 ± 0.98. Of the 143 studies, 115 studies received a score of 6–8, with 16 studies receiving a 5, 9 receiving a 4, and 2 studies receiving a 3 or less. Average scores by study type are as follows: Cohort = 6.4, Cross-Sectional = 6.2, Mixed Methods = 5.4, Non-Randomized Experimental = 6.4; Qualitative = 6, RCT = 5.7; Other = 4. The quality assessment for each study can be found in Appendix A.

## 4. Discussion

This systematic review provided a summary of the current evidence surrounding communication tools and approaches used to improve vaccine acceptance. Because most studies (>66%) demonstrated increased vaccine acceptance, these findings support implementation of communication strategies across healthcare settings. Communication strategies clustered around a few key themes: (1) system-level supports (ex: electronic health record reminders, standing orders) and reminder/recall interventions (ex: sending text message notifications), (2) patient education materials (ex: reframing of rationale for being vaccinated), and (3) provider training and tools (ex: scripts, prompts, communication framework. These strategies were utilized individually as well as collectively to enhance vaccine acceptance.

The first strategy identified in this review was system-level supports and reminder/call interventions, which was utilized across many settings and often included small changes to address the reasons behind vaccine hesitancy. The 5C psychological antecedents by Betsch and colleagues (2018) also may be a helpful framework. In brief, much of the behaviors surrounding vaccination relate to 5 key elements: confidence (patient beliefs or attitudes with regard to vaccines), complacency (their perceived risk associated with the vaccine-related disease), constraints (barriers experienced with vaccinations), calculation (their evaluation of the vaccine and associated information-searching), and collective responsibility (their perceptions of their responsibility of protecting others through vaccination) [172]. This framework has proven useful in understanding patient choices, particularly during the COVID-19 pandemic [13]. Multiple studies integrated electronic health record (EHR) prompts, automated reminder systems for patients and for providers, text messages, and reminder phone calls which increased vaccine conversations and vaccine acceptance [86,127,146,161]. Indeed, Krantz (2018) posited that the integration of reminder interventions sustained the increased vaccine acceptance rates [86]. Several other studies utilized community health workers and patient navigators to reach patients in their home and enhance appointment scheduling [120,143], which is another approach to expand access to vaccines. Many of these approaches addressed elements such as constraints and calculation as well as other elements of the 5C antecedents. Approaches across settings, whether primary care, hospitals, community pharmacies, or other care settings could be enhanced through small changes; each community can determine which approach(es) (text messages, community health workers, provider reminders, etc.) best reaches their patient population. However, some disadvantaged settings may have limited resources to incorporate these interventions, patients may lack reliable cell phones and text message access to receive notifications, or cultural considerations may alter the receptiveness of patients and providers to these interventions. This should be explored further and adapted appropriately within resource and cultural contexts. Additionally, patient educational materials, the second key strategy, could be another low-resource option that is transferable. Educational materials were often centered around a disease and addressing misinformation or reframing the discussion surrounding the “why” of becoming vaccinated [46,135,148], However, these materials are language- and cultural context-dependent and would need adapted considerably outside of the specific language and cultural contexts that align with each study.

Many of the studies included in this review integrated provider training, whether on the tools, behavioral-based communication strategies, or how to prompt vaccine conversations, as a foundational element, which was the third key strategy. Traditional health professions education has focused on a more presumptive, directive approach to discussing vaccines. Best practices have shifted to a collaborative, patient-centered approach to elicit underlying concerns and health beliefs surrounding vaccines [26]. Interventions that trained providers in MI consistently improved vaccine acceptance [19,131,133,168]. However, concerns surrounding vaccines can be deeper than merely confidence in a vaccine. For example, the Health Belief Model [173] to explore patient beliefs about a vaccine-preventable disease and their evaluation of risk and benefits while considering cues to action (such as knowing someone who experienced consequences of the disease) [14]. The Health Belief Model also posits that individuals’ perceived benefits, barriers, and self-efficacy influence their health behaviors. Approaches like MI operationalize these constructs by eliciting patients’ beliefs, resolving ambivalence, and strengthening personal motivation to vaccinate (addressing the benefits). The consistent success of MI across vaccine types and settings suggests that interventions grounded in behavior change theory are particularly effective.

Generally, interventions focused on improving vaccine confidence and acceptance were more effective when addressing adult vaccines versus childhood vaccines. This may be influenced by a variety of factors [174,175]. First, parents typically have a strong and healthy desire to protect their children from what they might perceive as unnecessary danger or risk. For example, some parents express concerns regarding the number of vaccine doses given to children based on recommended immunization schedules. Parents also may want to avoid the child receiving injections due to a fear of needles or medical personnel. Secondly, social media influences, mainstream media reports of vaccine-related illnesses, and community or religious beliefs regarding vaccines specifically in children, have significant influence or parental decisions regarding vaccines. Also, parents typically can make decisions about vaccines for themselves, while parents serve as surrogate decision-makers for their children. Thus, the analysis of the risks and benefits may change when one is making decisions on behalf of another person, particularly children who are more vulnerable. These factors likely influence the results of vaccine confidence and should be considered when communicating with parents [174,175].

Research also was conducted primarily in the outpatient setting, such as primary care offices, and over 50% of studies focused on physicians and nurses for the intervention. Given the regularity with which patients see a primary care provider for wellness visits and chronic care visits, this setting can offer opportunities to intervene. With significant shortages in primary care access within the United States [176] and even greater ones globally [177], the primary care setting cannot be the only point of contact for vaccine conversations. Other settings where interventions occurred included hospital settings and community pharmacies [22,37,40,69,84,104,117,118,124], which are access points for older adults, patients with limited insurance, or those in medically underserved areas. Patients visit their pharmacies nearly twice as often as their primary care provider [178], and many of the pharmacy-based interventions were successful in enhancing vaccine acceptance. For example, one community pharmacy intervention for patients who had previously declined an influenza vaccine resulted in 23% of patients accepting the vaccine [124]. Similarly, a hospital-based intervention for influenza resulted in nearly 40% more patients being vaccinated [156].

It also is important to consider the context of the interventions, as briefly alluded to with patient educational materials. While over 75% of the studies were conducted in the United States, a substantial portion were completed elsewhere, namely in Europe and Asia. Concerns surrounding vaccines exist within the United States, with declining vaccine acceptance in the core pediatric vaccine series [2,3]. Global perspectives on vaccines also have shifted, with gains in vaccine confidence that occurred in the late 2010s moving to declines in the 2020s [179]. Initially indicated as one of the most significant public health threats [180], declines in vaccine confidence and vaccine acceptance raise further concerns. Thus, given that reasons for vaccine confidence may vary geographically and culturally, broadening research settings is important. Given that over two-thirds of included studies were U.S.-based, generalization to low- and middle-income countries (LMICs) also should be made cautiously for all interventions, where structural and cultural determinants of vaccine confidence may differ substantially. As underscored in theories behind health behavior change, such as the Health Belief Model [173], vaccine confidence is shaped by modifying factors (ex: cultural norms, socioeconomic factors, trust in healthcare systems and providers) that may differ by geographic region [179,181,182]. Future research should, therefore, prioritize low- and middle-income settings and evaluate whether interventions are equally effective among marginalized populations, where access and trust deficits are often greater. Studies also covered many different vaccines; with influenza, HPV, and pneumococcal vaccines being the most commonly addressed. These findings may not apply to all vaccines in all settings; further research should explore the utility of these interventions in other vaccines (ex: COVID-19 vaccines), patient populations (ex: routine pediatric immunizations), and contexts (ex: LMICs, underserved areas). However, these vaccines have some of the largest public concerns about efficacy and necessity as well as safety as well as low acceptance [183,184,185]; which should be considered when intervening. Lastly, bundling of interventions should be a focus of future research, as these could have additive impacts on vaccine acceptance within various populations.

## 5. Limitations

Although a research librarian was utilized to refine the search strategy, articles meeting the inclusion criteria may have been missed and not included in the review, leading to selection and reporting bias. Due to the limitation of published research, evidence emerging from or existing outside of research may have been missed. Additionally, publication bias is possible, as studies focusing on vaccine confidence that produce negative results are less likely to be published. Self-reported measures of confidence or intention also may not translate directly to vaccine acceptance or uptake. Intervention fidelity, i.e., how consistently providers and health systems implemented communication techniques and approaches, were rarely measured, limiting the ability to link interventions with outcomes. Further, the predominance of U.S. studies limits external validity related to other parts of the world.

At each stage of the systematic review process, two researchers independently completed the step, with a third reviewer resolving discrepancies. However, the review process itself may have introduced errors and incorrectly excluded relevant studies. Due to heterogeneity across studies, a thematic synthesis was conducted, which may limit precise estimations of intervention effect. The lack of quantitative analyses underscores the variability across outcomes. Although most studies were of high methodological quality, a minority exhibited weaker design rigor, reducing the interpretability of their findings.

## 6. Conclusions

In summary, this systematic review highlights that provider–patient communication tools and approaches, particularly those emphasizing patient-centered, collaborative strategies, consistently improve vaccine acceptance. Research should continue to evaluate these findings across global settings, particularly in diverse sociocultural and sociopolitical settings as well as in settings with differ. While most interventions were studied in outpatient primary care, evidence also demonstrates the value of expanding communication and vaccination opportunities into pharmacies, hospitals, and other community-based settings to address gaps in access. The integration of behavioral communication strategies, such as MI, combined with provider training and system-level reminders, shows particular promise in improving vaccine acceptance. Further, healthcare settings can start small by providing tools to have better conversations, integrating reminder prompts into EHR systems, and ensuring patient engagement in the decision-making process. As vaccine confidence continues to decline globally, translating these findings into routine practice is critical to avoid the burden of communicable disease.

## Figures and Tables

**Table 1:** Overview of Included Study Characteristics.

Study Characteristic	All Studies *n* (%)	Adult Studies *n* (%)	Pediatric Studies *n* (%)
**Country**			
United States	110 (76.9)	66 (71.0)	60 (89.6%)
Europe	12 (8.4)	11 (11.8)	2 (3.0)
Asia	10 (7.0)	8 (8.6)	2 (3.0)
Canada	5 (3.5)	3 (3.2)	1 (1.5)
Africa	3 (2.1)	3 (3.2)	1 (1.5)
Australia	3 (2.1)	2 (2.2)	1 (1.5)
Study Design			
Experimental, non-RCT	59 (41.3)	36 (40.9)	33 (50.8)
RCT	52 (36.6)	31 (35.2)	18 (27.7)
Cross-Sectional	15 (10.6)	11 (12.5)	6 (9.2)
Mixed Methods	5 (3.5)	1 (1.1)	5 (7.7)
Cohort	5 (3.5)	5 (5.7)	---
Qualitative	4 (2.8)	2 (2.3)	1 (1.5)
Other	3 (2.1)	2 (2.3)	2 (3.1)
Setting			
Outpatient	112 (75.2)	70 (72.9)	53 (80.3)
Inpatient	12 (8.1)	6 (6.3)	6 (9.1)
Community Pharmacy	9 (6.0)	9 (6.0)	---
Other	16 (10.7)	12 (12.5)	7 (10.6)
Health Professions			
Physicians	100 (35.8)	57 (36.5)	56 (40.3)
Nurses	74 (26.2)	41 (26.3)	44 (31.7)
Medical Assistants	23 (8.2)	10 (6.4)	11 (7.9)
Pharmacists	20 (7.1)	12 (7.7)	6 (4.3)
Other Providers	12 (4.3)	6 (3.9)	5 (3.6)
Midwives	9 (3.2)	6 (3.9)	3 (2.2)
Residents	7 (2.5)	6 (3.9)	3 (2.2)
Medical Students	6 (2.1)	4 (2.6)	---
Fellows	2 (0.7)	2 (1.3)	1 (0.7)
Other	28 (9.9)	12 (7.7)	10 (7.2)
Vaccines Covered			
Influenza	60 (26.6)	44 (31.0)	21 (21.7)
HPV	55 (24.3)	23 (16.2)	40 (41.2)
Pneumococcal	27 (12.0)	21 (14.8)	2 (2.1)
Tdap	17 (7.5)	13 (9.2)	8 (8.3)
Core Pediatric Series	12 (5.3)	---	8 (2.25)
HBV	9 (3.9)	8 (5.6)	1 (1.0)
COVID-19	8 (3.5)	7 (4.9)	1 (1.0)
MCV	6 (2.7)	1 (0.7)	6 (6.2)
Shingles	6 (2.7)	6 (4.2)	---
Tetanus	5 (2.2)	4 (2.8)	2 (2.1)
HepA	4 (1.8)	3 (2.1)	1 (1.0)
Pertussis	4 (1.8)	2 (1.4)	1 (1.0)
MMR	2 (0.9)	1 (0.7)	1 (1.0)
Diphtheria	2 (0.9)	1 (0.7)	1 (1.0)
Polio	1 (0.4)	---	1 (1.0)
Rotavirus	1 (0.4)	---	2 (2.1)
Varicella Zoster Virus	1 (0.4)	1 (0.7)	2 (2.1)
BCG	1 (0.4)	---	---
Other	5 (2.2)	6 (4.2)	---
Intervention Type			
Communication Approach	103 (72.0)	65 (51.2)	54 (53.5)
Communication Tool	103 (72.0)	62 (48.8)	47 (46.5)

Key: RCT = randomized controlled trial; HPV = human papillomavirus; Tdap = tetanus, diphtheria, and acellular pertussis; HBV = hepatitis B virus; MCV = meningococcal conjugate vaccine; HepA = hepatitis A; MMR = measles, mumps, and rubella; BCG = Bacillus Calmette-Guérin.

**Table 2:** Core Themes Regarding Communication Tools and Approaches Utilized.

Themes	Count	Examples
Provider-Focused Training and Tools	47	“Ask, Acknowledge, Advise” communication training [76] CME-based education on vaccines [58] In-person quality improvement training or remote provider communication training (Announcement Approach Training (AAT)) [71] Training on a script for patient encounters [51]
Patient Education Strategies	47	Gain-framed messages, loss-framed messages, flyers [46] Cancer prevention booklet reframing HPV vaccine as cancer prevention, not STI prevention [148] Educational videos (4 versions) and a bilingual flyer (summarize information regarding HPV vaccine, prompt questions) [135]
Provider–Patient Communication Strategies	46	Motivational interviewing (MI) [131,133,168] Provider tool with efficacy/safety/religion themes to guide vaccine conversations using an MI framework [19] Participatory communication styles [141]
System-Level and Structural Supports	23	Standing orders for vaccines [50,125,129] Provider prompts to discuss vaccines [42,106,125,129]
Reminder and Recall Interventions	22	Trained community health workers supported appointment scheduling through in-home visits [120] Patient navigator conducted reminder calls [143] Reminder phone calls for parent consent return for HPV vaccine [161]

CME = Continuing Medical Education; HPV = human papilloma virus; MI = motivational interviewing; STI = sexually transmitted infection; AAT = Announcement Approach Training.

**Table 3:** Impact of Tool or Approach on Vaccinations.

Outcome	All Studies *n* (%)	Adult Studies *n* (%)	Pediatric Studies *n* (%)
Positive: Improvement in vaccine acceptance	89 (66.4)	61 (45.5)	44 (32.8)
Negative: Decline in vaccine acceptance	1 (0.7)	1 (0.7)	0 (0.0)
Neutral: Limited or no change in vaccine acceptance	7 (5.2)	3 (2.2)	4 (3.0)
Mixed findings: Positive and some negative vaccine acceptance	6 (4.5)	2 (1.5)	5 (3.7)
Mixed findings: Positive and some neutral vaccine acceptance	29 (21.6)	15 (11.2)	19 (14.2)
Mixed findings: Positive, negative, and neutral vaccine acceptance	2 (1.4)	1 (0.7)	1 (0.7)

## Data Availability

The raw data supporting the conclusions of this article will be made available by the authors on request.

## Appendix A. Mixed Methods Assessment Tool (MMAT) Evaluation of Study Quality

**Table A1:** Mixed Methods Assessment Tool (MMAT) Study Quality.

Author (Year)	MMAT Part I Score ^1^	MMAT Part II Score ^2^	Total Score ^3^
**Cohort Studies**			
Brackett (2015)	2	5	7
Lim (2022)	2	5	7
Schirwani (2022)	2	4	6
Shafer (2021)	2	4	6
Villaverde Piñeiro (2024)	2	4	6
**Cross-Sectional Studies**			
Fallucca (2023)	2	5	7
Omer (2018)	2	5	7
Suryadevara (2016)	2	5	7
Warner (2020)	2	5	7
Desai (2021)	2	4	6
Gilkey (2016)	2	4	6
Hofstetter (2017)	2	4	6
Ludwikowska (2020)	2	4	6
Margolis (2021)	2	4	6
Moss (2016)	2	4	6
Opel (2015)	2	4	6
Sato (2022)	2	4	6
Vu (2022)	2	4	6
Thompson (2021)	2	3	5
**Mixed-Methods Studies**			
Berenson (2019)	2	5	7
Cox (2022)	2	4	6
Jain (2015)	1	4	5
Kempe (2014)	2	3	5
Tabana (2016)	2	2	4
**Non-Randomized Experimental Studies**			
Baltes (2022)	2	5	7
Bar-Shain (2015)	2	5	7
Bechini (2019)	2	5	7
Bluml (2018)	2	5	7
Brewer (2021)	2	5	7
Caffrey (2018)	2	5	7
Cates (2018)	2	5	7
Cebollero (2020)	2	5	7
Chou (2014)	2	5	7
Clark (2015)	2	5	7
Cole (2022)	2	5	7
Davis (2022)	2	5	7
Dehlinger (2021)	2	5	7
Fenton (2021)	2	5	7
Foradori (2020)	2	5	7
Frew (2022)	2	5	7
Gattis (2019)	2	5	7
Kaufman (2020)	2	5	7
Krantz (2018)	2	5	7
Loskutova (2020)	2	5	7
Molokwu (2023)	2	5	7
Nowalk (2012)	2	5	7
Page (2020)	2	5	7
Pati (2015)	2	5	7
Perkins (2015)	2	5	7
Polonijo (2021)	2	5	7
Rand (2018)	2	5	7
Sanderson (2017)	2	5	7
Zhang (2023)	2	5	7
Adam (2015)	2	4	6
Connors (2018)	2	4	6
Cotugno (2017)	2	4	6
Dehnen (2019)	2	4	6
Freedman (2015)	2	4	6
Fu (2017)	2	4	6
Hirshberg (2021)	2	4	6
Kositzke (2023)	2	4	6
Lecce (2021)	2	4	6
Li (2019)	2	4	6
Mazzoni (2016)	2	4	6
McLean (2017)	2	4	6
Queeno (2017)	2	4	6
Rand (2018)	2	4	6
Rao (2018)	2	4	6
Rao (2020)	2	4	6
Salous (2020)	2	4	6
Sandokji (2022)	2	4	6
Sobota (2015)	2	4	6
Stetson (2019)	2	4	6
Stevenson (2000)	2	4	6
Strasel (2024)	2	4	6
Suryadevara (2019)	2	4	6
Townsend (2018)	2	4	6
Wedel (2016)	2	4	6
Wright (2019)	2	4	6
Zacharias (2015)	2	4	6
Falcone (2020)	2	3	5
McCarthy (2015)	2	3	5
Labbe (2022)	2	2	4
Suryadevara (2021)	2	1	3
**Qualitative Studies**			
Lwembe (2016)	2	5	7
McGinnis (2021)	2	5	7
Shay (2016)	2	4	6
Todorova (2014)	2	2	4
**Randomized Controlled Trials (Rcts)**			
Dempsey (2018)	2	5	7
Dudley (2022)	2	5	7
Goodman (2015)	2	5	7
Lau (2012)	2	5	7
Lockhart (2018)	2	5	7
Rand (2017)	2	5	7
Scott (2019)	2	5	7
Chan (2015)	2	5	7
Chamberlain (2015)	2	4	6
Debroy 2023	2	4	6
Ekmez (2022)	2	4	6
Esposito (2009)	2	4	6
Fisher (2023)	2	4	6
Fisher-Borne (2018)	2	4	6
Gilkey (2022)	2	4	6
Gilkey (2023)	2	4	6
Ginson (2000)	2	4	6
Hanley (2023)	2	4	6
Henrikson (2015)	2	4	6
Kasting (2019)	2	4	6
Klassing (2018)	2	4	6
Kriss (2017)	2	4	6
Launay (2014)	2	4	6
Meharry (2014)	2	4	6
Milkman (2021)	2	4	6
Milkman (2022)	2	4	6
Muñoz-Miralles (2022)	2	4	6
Nowalk (2016)	2	4	6
Ozdemir (2023)	2	4	6
Patel (2023)	2	4	6
Perkins (2020)	2	4	6
Reno (2018)	2	4	6
Reno (2019)	2	4	6
Werk (2019)	2	4	6
Wong (2016)	2	4	6
Brewer (2017)	2	3	5
Coenen (2017)	2	3	5
**Other**			
O’Donnell (2018)	2	4	6
Whelan (2014)	2	3	5
Wolynn (2023)	0	1	1

^1^ MMAT Part I Scores are based on two items surrounding clear research questions and addressing the research questions. Higher scores indicate greater study quality, with scores ranging from 0–2. ^2^ MMAT Part II Scores are based on the methodology of the study, such as the approach; analysis; randomization, blinding, and adherence (RCTs); representativeness; measurements. Higher scores indicate greater study quality, with scores ranging from 0–5. ^3^ Higher scores indicate greater study quality, with scores ranging from 0 to 7.
